# Supplementary material for: Volumetric atlas of the rat inner ear from microCT and iDISCO+ cleared temporal bones
Source: PeerJ. 2025 May 26;13:e19512. doi: 10.7717/peerj.19512 (PMC12121623; doi:10.7717/peerj.19512)
Supplement: Supplemental Information 4 — The inner ear is divided in Cochlea and Vestibular Apparatus, following (Osen et al., 2021). Each subdivision is divided into bony labyrinth, membranous labyrinth, sensorineural and other structures. Arteries and bones are classified in a separate "other" category. [file peerj-13-19512-s004.docx]

| **Inner ear hierarchy** | | | | |  | **Abbreviation** |
| --- | --- | --- | --- | --- | --- | --- |
| **Inner ear** | | | | | | IE |
|  | **Cochlea** | | | | | Co |
|  |  | **Cochlear bony labyrinth** | | | |  |
|  |  |  | Scala tympani | | | Co,st |
|  |  |  | Scala vestibuli | | | Co,sv |
|  |  |  | Round window membrane | | | Co,rwm |
|  |  | **Cochlear membranous labyrinth** | | | |  |
|  |  |  | Scala media | | | Co,sm |
|  |  | **Cochlea, sensorineural** | | | |  |
|  |  |  | **Cochlear nerve** | | | 8cn |
|  |  |  |  | Cochlear nerve (central) | | 8cn,c |
|  |  |  |  | Cochlear nerve (peripheral) | | 8cn,p |
|  |  |  |  | Cochlear nerve (dendrite) | | 8cn,d |
|  |  |  |  | Spiral ganglion | | 8cn,SpG |
|  |  |  | **Cochlear sensory epithelium** | | |  |
|  |  |  |  | Organ of Corti | | Co,OoC |
|  |  | **Cochlea, other** | | | |  |
|  |  |  | Stria vascularis | | | Co,sva |
|  |  |  | Spiral ligament | | | Co,slg |
|  |  |  | Spiral limbus | | | Co,slm |
|  | **Vestibular apparatus** | | | | | VeA |
|  |  | **Vestibular bony labyrinth** | | | |  |
|  |  |  | Vestibular perilymph | | | VeA,p |
|  |  | **Vestibular membranous labyrinth** | | | |  |
|  |  |  | Saccule | |  | VeA,sac |
|  |  |  | Utricle | |  | VeA,utr |
|  |  |  | Semicircular canals | |  | VeA,scc |
|  |  |  | Endolymphatic duct | |  | VeA,ed |
|  |  |  | Reuniting duct | |  | VeA,rd |
|  |  | **Vestibule, sensorineural** | | | |  |
|  |  |  | **Vestibular nerve** | | | 8vn |
|  |  |  |  | Superior vestibular nerve | | 8vn,s |
|  |  |  |  | Inferior vestibular nerve | | 8vn,i |
|  |  |  | **Vestibular sensory epithelium** | | |  |
|  |  |  |  | Saccular macula | | VeA,msa |
|  |  |  |  | Utricular macula | | VeA,mut |
|  |  |  |  | Posterior canal crista | | VeA,pcc |
|  |  |  |  | Anterior canal crista | | VeA,acc |
|  |  |  |  | Lateral canal crista | | VeA,lcc |
|  |  | **Vestibule, Other** | | | |  |
|  |  |  | Saccular connective tissue | | | VeA,csa |
|  |  |  | Membrana limitans | | | VeA,ml |

| **Other inner ear related structures** | Abbreviation |
| --- | --- |
| Cochlear artery | Art,co |
| Vestibular artery | Art,ve |
| Stapedial artery | Art,sp |
| Temporal bone | Tbo |
| Stapes | Sp |
