# Supplementary figures and images for: Volumetric atlas of the rat inner ear from microCT and iDISCO+ cleared temporal bones
Source: PeerJ. 2025 May 26;13:e19512. doi: 10.7717/peerj.19512 (PMC12121623; doi:10.7717/peerj.19512)

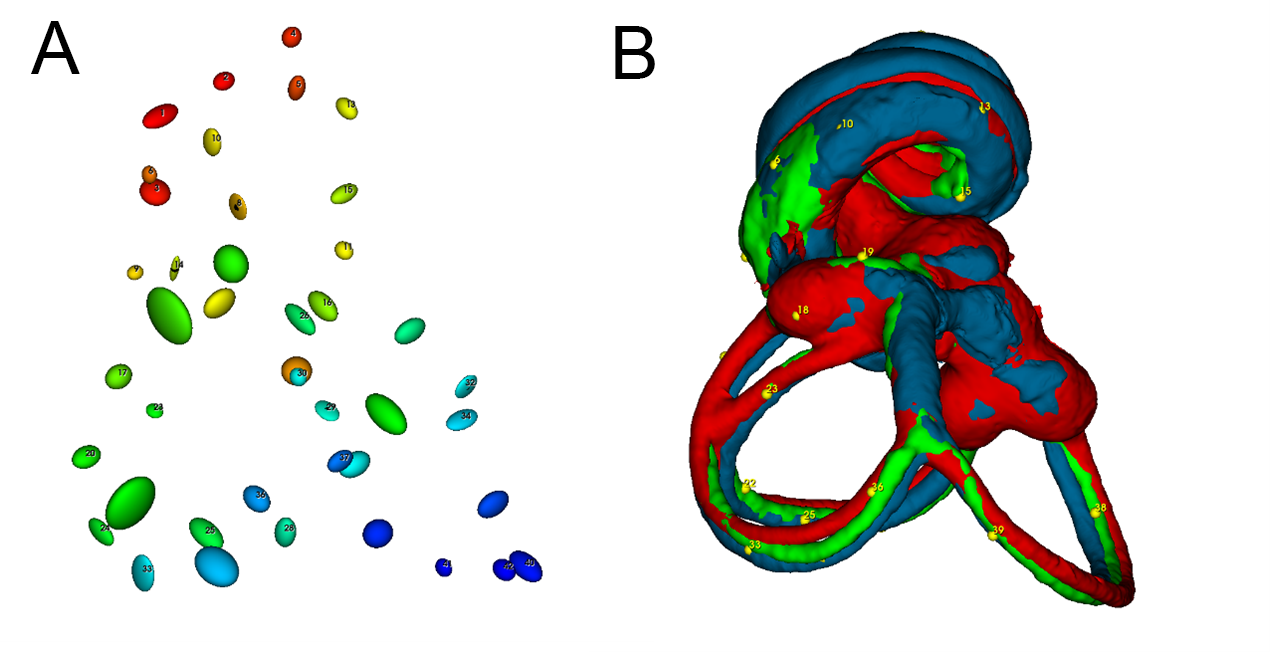

Supplement: Supplemental Information 5 — A: Ellipsoids showing Procrustes distances (magnified 5x) for each landmark placed on the labyrinths. B: Superposition of bony labyrinth volumes from microCT and lightsheet samples after rigid transformation (scaling, rotation, translation) for maximal congruence with ALPACA. [file peerj-13-19512-s005.png]

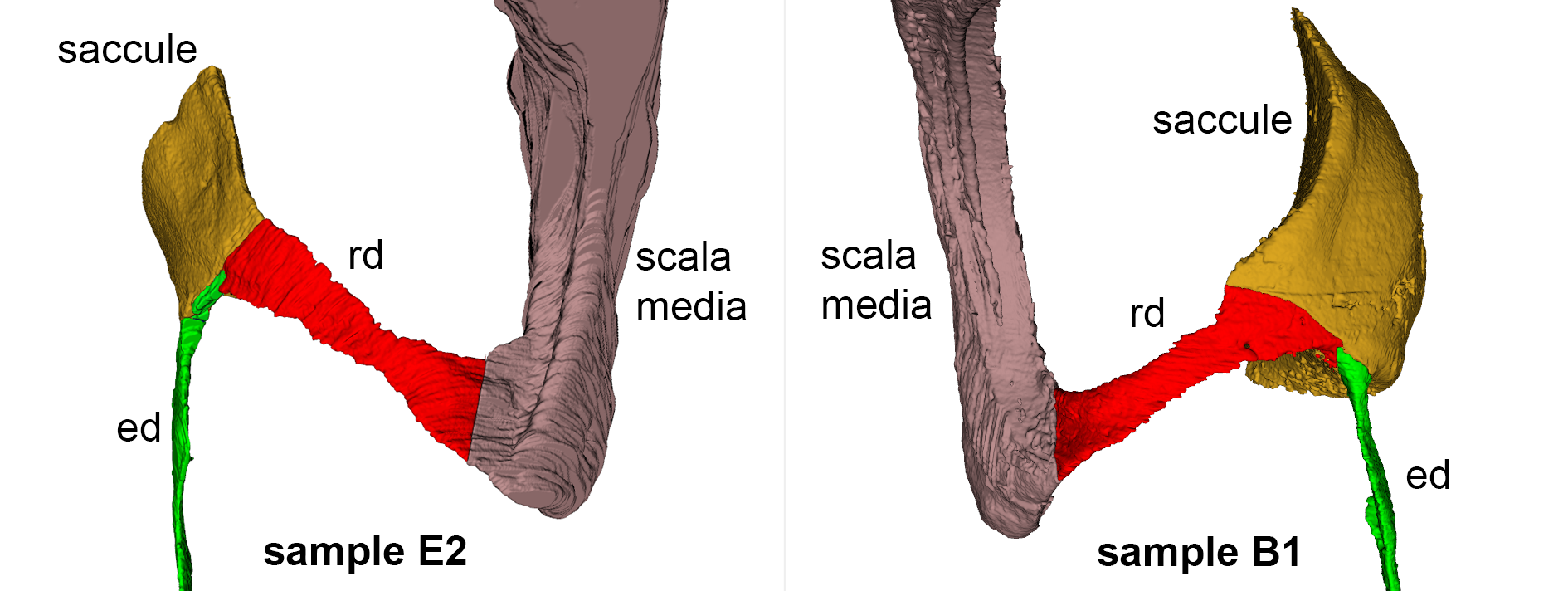

Supplement: Supplemental Information 6 — The saccule in rat E2 was only partially visible, and its reconstruction is incomplete. Rd: reuniting duct; ed: endolymphatic duct [file peerj-13-19512-s006.png]

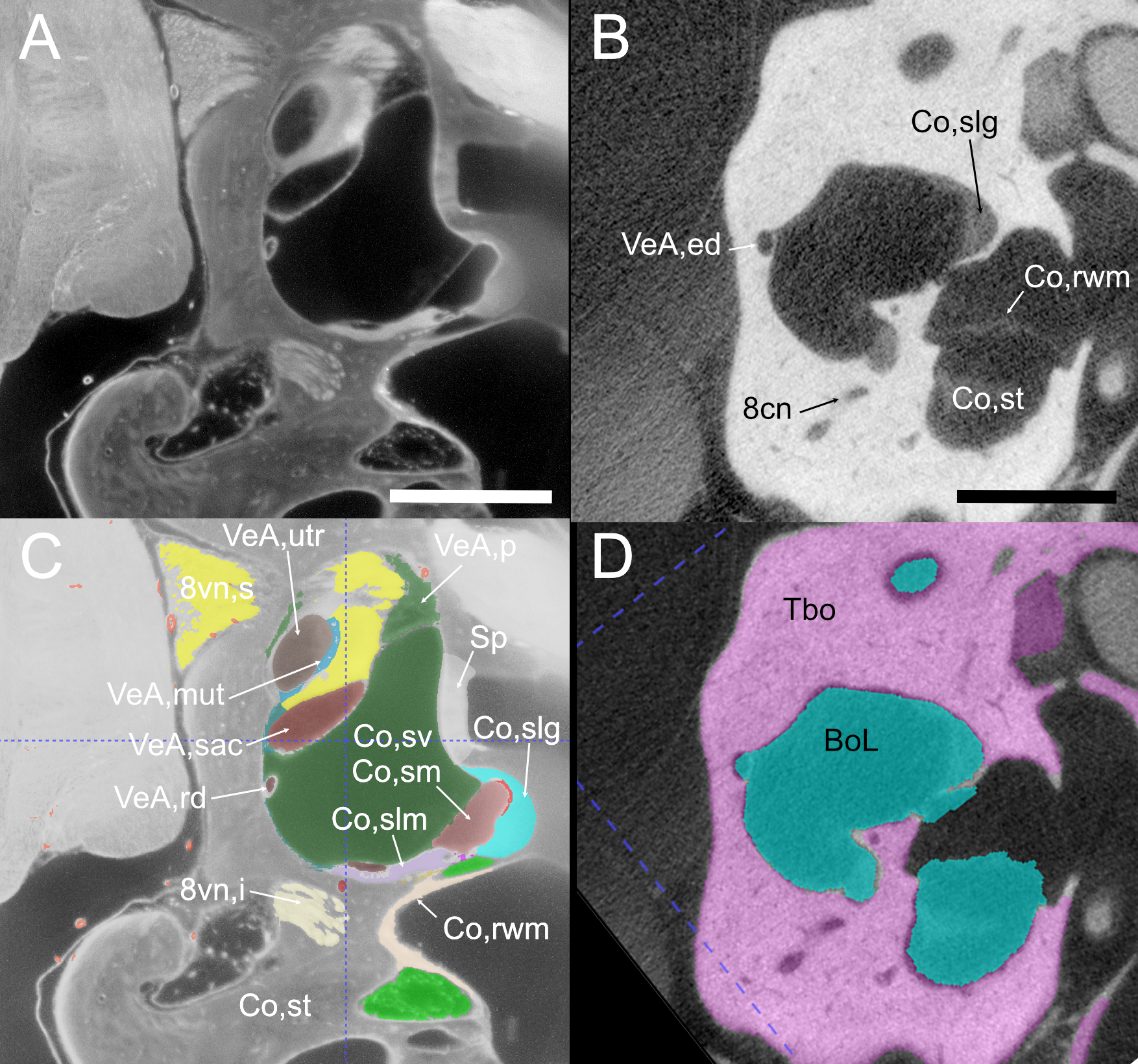

Supplement: Supplemental Information 7 — A: single optical section of Rat B showing autofluorescence signal. Scale bar: 1 mm. B: single section of microCT stack displaying bone and several soft tissue elements, such as the round window. Scale bar: 1 mm. C, D: same as A, B but with annotated segmentations added. 8cn: cochlear nerve, 8vn: vestibular nerve (i: inferior, s: superior), BoL: bone labyrinth, Co: cochlea (rwm: round window membrane, slg: spiral ligament, slm: spiral limbus, sm: scala media, st: scala tympani, sv: scala vestibuli), Sp: stapes, TBo: temporal bone, VeA: vestibular apparatus (ed:endolymphatic duct, mut: macula utriculi, p:perylimph, rd: reuniting duct, sac: saccule, utr: utricle). [file peerj-13-19512-s007.png]
